# Historical Study for the Differences of Processing of *Pinellia ternata* Tuber Between China and Japan

**DOI:** 10.3389/fphar.2022.892732

**Published:** 2022-06-20

**Authors:** Yan Liu, Misato Ota, Tsukasa Fueki, Toshiaki Makino

**Affiliations:** ^1^ Department of Pharmacognosy, Graduate School of Pharmaceutical Sciences, Nagoya City University, Nagoya, Japan; ^2^ Matsuya Pharmacy, Niigata, Japan; ^3^ Department of Traditional Medicine, Toho University School of Medicine, Tokyo, Japan

**Keywords:** *Pinellia ternata* tuber, processing, history, traditional Chinese medicine, traditional Japanese kampo medicine

## Abstract

Pinellia Tuber (the dried tuber of *Pinellia ternata* (Thunb.) Makino [Araceae]) (PT) is a crude drug used in traditional Chinese medicine (TCM) and Japanese Kampo medicine. PT is subjected to additional processing before use in TCM because of its toxic, while the processing has not been used in Kampo medicine. The aim of this study is to clarify the reason why the differences about the processing of PT between TCM and Kampo medicine have been appeared. We investigated successive literatures published in China and in Japan from the Han dynasty to the modern age. The descriptions about the processing of PT in China had appeared since the Later Han dynasty as washing, and after that, various processing methods have been recorded, such as boiling, steaming, making cakes, and fermenting to prepare PT malt (PTM) with various drug additives. The objective of the processing for PT was not only to remove its toxicity but to change drug properties, and several kinds of processed PT had been developed to treat different types of “phlegm” in the Ming dynasty. The current Chinese Pharmacopoeia recommends the use of processed PT to avoid the toxicity, and registers unprocessed PT as well as three kinds of processed PT except for PTM which had been deleted in 2015 edition. These processing methods for PT have been established in the Qing dynasty. The oldest description in Japan was appeared in 1363, and the processing methods had been influenced by the literatures in the Song dynasty. After that, the processed PT in Japan had mainly been PTM until the 18th century. In 1738, Shuan Kagawa wrote that PT should not be processed because its pharmacological effects disappeared and the toxicity of PT disappeared by preparing its decoction without processing. Then, the processing of PT has been unpopular, and the Japanese Pharmacopoeia has registered PT since 1939 without any processing. Compared to TCM, Japanese Kampo medicine has tended to avoid ideologism based on traditional knowledge and to adopt positivism. This policy has reflected the differences in the processing of PT between Kampo medicine and TCM.

## Introduction

Pinellia Tuber (the dried tuber of *Pinellia ternata* (Thunb.) Makino [Araceae], 半夏, PT) has a long history of clinical application in China. It was first recorded in *Shen Nong’s Herbal Classic*, published approximately 2000 years ago (Wang, 2010). In traditional Chinese medicine (TCM), PT has expectorant and anti-nausea effects for the management of cough, *phlegm*, vomiting and cancer (Bensky et al., 2004). However, it has also been stated that PT has “toxicity” since the ancient TCM literature *Miscellaneous Records of Famous Physicians* published around 2,000 years ago (Tao, 2013). Indeed, when raw PT is orally administered, symptoms of sharp tingling in the lips and pain in the mouth and throat are apparent. A previous study indicated that this strong irritation has been attributed to the needle-like crystals of calcium oxalate, called raphides. Indeed, these raphides have been considered as the possible toxic substances of raw PT (Fueki et al., 2016). As a result, the processing methods of raw PT for detoxification have been developed in TCM, although the mechanisms of raw PT-induced toxicity are still not fully understood. The current Chinese Pharmacopoeia 2020 edition states that raw PT is toxic, and it should only be used for internal use after processing. In this Pharmacopoeia, three processed PT items are registered, Prepared PT (法半夏, PPT), PT prepared with ginger (姜半夏, GPT), and PT prepared with alumen (清半夏, APT). PPT is produced by soaking PT in a decoction of licorice root with lime solution with a pH over 12 until the cut surface is uniform yellow. GPT is produced by adding PT and alumen to a decoction of ginger slices and thoroughly boiling the mixture. APT is produced by soaking PT in an 8% solution of alumen until the center of the cut surface without a dry core and with a slight-numbing taste (Chinese Pharmacopoeia Commission, 2020). Various experiments have been carried out on the toxicity reduction by these processes (Zhong, 2007; Jin et al., 2019). During the processing of PPT, immersion in licorice juice and limewater can destroy the structure of the raphides and reduce the amount of lectin protein (Li et al., 2020). Processing with ginger causes structural changes in the raphides (Wu et al., 2010; Fueki et al., 2020), and its active ingredient in ginger was identified as oxalic acid (Fueki et al., 2022). It has also been suggested that gingerol from ginger juice can effectively inhibit raw PT-induced inflammation (Yu H. et al., 2015). During the process of APT, Al^3+^ in the alumen solution forms a complex with the oxalate radical in calcium oxalate crystals, which promotes the destruction and dissolution of calcium oxalate and destroys the structure of the poison needle crystal. The generated Al(OH)_3_ colloid adsorbs and agglutinates the toxic components of PT (Yu H. L. et al., 2015). Nevertheless, up to the present, the underlying toxicity-reducing mechanisms of processing are uncertain.

By contrast, the Japanese Pharmacopoeia 18th Edition published in 2021 registers only unprocessed PT and does not register any processing methods for PT (The Society of Japanese Pharmacopoeia, 2021). Among 297 items of the OTC Kampo medicinal products and 148 items of ethical Kampo extract formulations approved by the authority of the Medicines Regulatory Agency in Japan (Japan Kampo Medicines Manufacturers Association, 2012; 2013), processed PT is never been prescribed as a component in Kampo formulations. Therefore, there is no practice to process PT in Japanese traditional Kampo medicine, and PT is not recognized as a toxic crude drug in Japan at present, although some records of processed PT do exist in old Japanese medicinal literatures.

In China, the history of PT processing has already been reviewed in several studies (Chi, 1989; Wu, 2001; Xiu et al., 2006; Zhang et al., 2015a; Na et al., 2017; Li et al., 2019; Zhao et al., 2020). However, the changes in processing methods and their objectives according to the successive literature have not been discussed well. Furthermore, we cannot find historical studies on the processing of PT in Japan and the differences in processing of PT between mainland China and Japan. The present study reveals that the successive medical and medicinal literature has seldom described about PPT and APT in old medical and medicinal literatures in both China and Japan, and that among several kinds of processed PT products, the most common was PT malt (半夏曲, 半夏麯, PTM), a fermented dumpling prepared from powdered PT with ginger juice, in old Japanese medicinal literatures. PTM has been registered in previous Chinese Pharmacopoeia, but not in the current edition (Chinese Pharmacopoeia Commission, 2020). PTM appeared in the literature published during the Song dynasty, and its history was reviewed by Zhang et al. (Zhang et al., 2015a). However, they did not discuss the transition of the substance that the term PTM had referred to, how to distinguish the usages or PTM from other processed PT products, why the current Chinese Pharmacopoeia has deleted PTM, and why PTM has fallen from use in Japanese Kampo medicine. We investigated and compared the successive medical and medicinal literature in mainland China and Japan to pick up the descriptions about the processing of PT, reviewed them, and discussed how past physicians and pharmacists distinguished PTM among processed PT products and the reason that PTM is not used in current Kampo medicine.

## Materials and Methods

We investigated medicinal and medical literature published in mainland of China and in Japan from the Later Han dynasty to the modern age using the databases of *Guoxuemi* http://www.guoxuemi.com, *Zhongyishijia* http://www.zysj.com.cn, and the introduction to old books in Chinese medicine https://www.theqi.com/cmed/oldbook/index.html, National Diet Library Digital Collections https://dl.ndl.go.jp, Waseda University Library’s collection of Japanese and Chinese classics https://www.wul.waseda.ac.jp/kotenseki/index.html, Kyoto University Rare Materials Digital Archive https://rmda.kulib.kyoto-u.ac.jp/en, We also investigated the successive editions of Chinese Pharmacopoeias published from 1953 to 2020 (Chinese Pharmacopoeia Commission, 2020), and of Japanese Pharmacopoeia published from 1886 to 2021 (The Society of Japanese Pharmacopoeia, 2021), and personal belongings. Next, we searched for phrases about the processing of PT and confirmed the existence of these phrases in the books registered in the library, or personally held articles. English terms in TCM and Kampo medicine were used according to Bensky’s reference book (Bensky et al., 2004), standard published by World Federation of Chinese Medicine Societies (World Federation of Chinese Medicine Societies, 2007), and the dictionary published by the Japan Society of Oriental Medicine (The Editing Committee for Dictionary of Kampo Medicine the Japan Society for Oriental Medicine, 2020). When the titles of the literatures are not translated in English in these reference books, the titles are described in English letters of pinyin or Japanese romanized spelling.

## Results

### Descriptions of PT Processing in Successive Medical Literatures Published in Mainland China

Descriptions of the processing of PT in the successive medical formulary literature written by physicians, describing the usage of the medicinal substances to make formulas for use in clinics published in mainland China are shown in Supplementary Table S1. The first descriptions of the processing of PT appeared during the Han dynasty. *Treatise on Cold Damage* and *Synopsis of the Golden Chambe*r described the use of PT after washing and after boiling, respectively (Zhang, 2000). The former also describes the toxicity of PT. *Classics of Golden Chamber and Jade Case* described that PT should be washed with hot water well to remove its toxicity (Zhang, 2009). In the Eastern Jin dynasty, the *Handbook of Prescriptions for Emergency* described that PT should be washed with hot water five to six times, and this operation was named as “wash-processing (熟洗)" in other formulas (Ge, 2016). This was the first literature describing about the processing of making PT cake. Meanwhile, the first description of PTM appeared in *Huatuo’s devine formulations* (Hua, 2010); however, there is no description of the preparation method of PTM in this literature.

In the Northern and Southern dynasties, the processing method of soaking with ginger juice was first described in *Liu Juanzi’s Ghost-Bequeathed Prescriptions* published in 499 (Liu, 1970). In the Tang dynasty, the *Supplement to Prescriptions Worth a Thousand Gold Pieces* published in 682 (Sun, 1982; Tao, 1997) described that since the needles would stuck at the throat, it was necessary to prepare PT by washing off the viscous skin, and using ginger was better as a drug adjuvant. This literature also described liquor as a drug adjuvant for PT.

During the Song dynasty, the item name of processed-PT (熟半夏) first appeared in the *Formulary of the Bureau of Taiping People’s Welfare Pharmacy* (Taiping People’s Welfare Bureau, 1975). This literature first described the preparation method of PTM, and registered the formula called “New-prepared-PT decoction” using roasted PTM. This literature also described several kinds of processed PT and first used alumen and foxtail millet as drug adjuvants for the processing of PT. In the *Comprehensive Recording of Sage-like Benefit* published in 1117, PTM was prepared using PT powder with ginger (North Song Imperial Government, 1962). In this literature, *Polyporus umbellatus* sclerotial, *Morus alba* root cortex, bran, rice, and vinegar were newly used as drug adjuvants for the processing of PT. *Bianque’s Heart Book*, published in 1146 (Dou, 2016), and *Chuanxinshiyongfang*, published in 1180, first used *Gleditsia sinensis* hulls, which contains saponins, and radish as drug adjuvants for the processing of PT, respectively. *One Hundred Questions about Woman’s Diseases* published in 1220 (Qi, 2012) described the mashing of PT with ginger for the preparation of PTM.

In the Yuan and Ming dynasties, *Danxi’s Experiential Therapy*, published in 1347, first used the slop from rinsing rice and sesame oil as drug adjuvants for the processing of PT (Zhu, 1347). *Han’s General Medicine*, published in 1522, described several preparation methods for PTM (Han, 1522). This literature describes the method of wrapping with the leaves of *Broussonetia* sp. and used bamboo sap and *Vitex negundo* var. *cannabifolia* stem juice as drug adjuvants for the processing of PT for the first time. The preparation method of PTM also changed in this literature to ferment dumplings made from powdered PT with ginger or alumen. Furthermore, this literature described the differences in the efficacies of PTM products using different drug adjuvants for the first time. Specifically, for *wind phlegm*, *Gleditsia sinensis* hulls, for *fire phlegm*, ginger juice with bamboo sap or *Vitex negundo* var. *cannabifolia* stem juice, for *moist phlegm* and *white cold phlegm*, concentrated ginger decoction with alumen before making PTM. The *Compendium of Medicine*, published in 1565, first used *Croton tiglium* seeds as a drug adjuvant for the processing of PT (Lou, 1565).

In 1587, the *Restoration of Health from the Myriad Diseases* first used the term PPT as the item name of PT after processing with cold water containing lime, alumen, and mirabilite without using hot water and ginger (Gong, 2013). In the medical literature published in the 17th century, PTM was prescribed in some formulas (Li, 1637; Wang, 1977). In 1759, *Huizhitangjingyanfang* recorded two items of PPT and PTM, and various drug adjuvants were used for the processing of PPT (Tao, 1759). In this literature, flour used as drug adjuvant was the first to appear in the successive medical literature in mainland China.

In the 18th century, *Shenxianjishiliangfang*, published in 1797 (Bai, 2005), recorded nine formulas prescribed with PPT and one formula prescribed with PTM. The first appearance of the term GPT as the item name was recorded in *New Compilation of Proved Prescriptions*, published in 1846 (Bao, 2007), which recorded 14 formulas prescribed with PPT, nine formulas prescribed with GPT, and three formulas prescribed with PTM. Notably, the item name APT had not been appeared in the successive medical literature before the 19th century.

### Descriptions About PT Processing in the Successive Medicinal Literatures Published in Mainland China

Descriptions of the processing of PT in the medicinal literature written by naturalists covering the characteristics of herbal medicinal substances published in mainland China are shown in Supplementary Table S2.

The first description of the processing of PT appeared in *Miscellaneous Records of Famous Physicians* published around 2,000 years ago, describing the washing of PT with hot water to remove the viscous skin (Tao, 2013). *Collective Commentaries on Classics of Materia Medica* (Chen and Wu, 2013), published during the Northern and Southern dynasties, described that since PT caused irritation at the throat, it was necessary to prepare them by washing off the viscous skin, and adding ginger was necessary to reduce the toxicity. In the Sui and Tang dynasties, ginger, liquor, white mustard, and vinegar were used as drug adjuvants (Zhen, 1983; Lei, 2010; Su, 2013). In the Ming dynasty, alumen and licorice root were used as drug adjuvants (Xue, 2015) for the processing of PT.

*Compendium of Materia Medica* published in 1552–1578 first arranged the item names of processed PT described in medical literature as “PT powder (半夏粉)," “PT cake (半夏餅)," and PTM (Li, 2004). “PT powder” was prepared by soaking powdered PT in hot water containing ginger juice for 3 days and being dried. “PT cake” was prepared by mixing powdered PT with ginger juice to make cake and being dried. PTM was prepared by mixing powdered PT with ginger juice and alumen to make dumplings, wrapping them with the leaves of *Broussonetia* sp., and fermenting them until yellowish malt flowers appeared. *Compendium of Materia Medica* also described new drug adjuvants for the processing of PT using *Gleditschia japonica* hull, bamboo sap, the juice of *Vitex negundo* var. *cannabifolia* stem, the slope from rinsing rice, and white lead frost. In this literature, the item names of “PT powder” and “PT cake” were the first appearance among both medical and medicinal literatures published previously, and this literature contained the first appearance of PTM in the medicinal literature.

From the middle of the 16th century to the middle of the 17th century, descriptions of the processing of PT did not change. The *Enlightneing Primer of Materia Medica*, published in 1560 (Chen, 2013), *Illumination of Materia Medica*, published in 1578 (Huang, 2011), *Penetrating the Mysteries of Materia Medica*, published in 1655 (Li, 2015), and *Treasury of Letters on Materia Medica*, published in 1666 (Gu, 2015), described only PTM as the processed PT product.

*Encountering with Origin of Herbal Classic*, published in 1695 (Zhang, 2011), and *Liangpenghuiji*, published in 1711 (Sun, 1711), first used the bile of pigs and *Notopterygium incisum* rhizomes as drug adjuvants for the processing of PT, respectively.

In 1765, *Supplement to Compendium of Materia Medica* used the new item “Mountain Hermit’s PT (仙半夏)" as one of the PTM products that had the best efficacy to remove *phlegm*, derived from the description of PPT in *Restoration of Health from the Myriad Diseases*, published in 1587 (Zhao, 1983), with slight modification. This literature also referred to PTM in the *Compendium of Materia Medica* as “PPT,” however, it described that several kinds of PTM products, some of which were called “Mountain Hermit’s PT” sold in drug stores were different from the original PPT. *Seeking Accuracy in Materia Medica*, published in 1769 (Huang, 1979), and *Delving into the Description of Materia Medica*, published in 1833 (Yang, 1958), also described several kinds of PTM products using various drug additives. *Harm and Benefit in Materia Medica*, published in 1862, recorded two items of PPT and PTM (Zhou, 2012). In this literature, the activity of PPT was weak, and PTM was better at removing *phlegm*.

The objective for the processing of PT was first described as a reduction in the toxicity of PT in 536 (Chen and Wu, 2013), and was published in 600 (Zhen, 1983), in 659 (Su, 2013), in 1116 (Chen and Wu, 2013), in 1624 (Zhang, 1624), in 1769 (Huang, 1979), and in 1828 (Zhang, 2013). In 1248, the different efficacies of PT and processed PT products for the treatment of vomiting and diarrhea first appeared in *Materia Medica for Decoctions* (Wang, 2008). This literature also described that by processing with ginger, PT could be used to remove *phlegm* and drool, open the *stomach*, and strengthen the *spleen*. The differences in the efficacies of different *phlegm* have been described in several kinds of PTM depending on the drug adjuvants, similar to the medical literature, which first appeared in *Compendium of Materia Medica* (Li, 2004). In this era, the literature described that the efficacies of raw PT were sharper than those of PTM (Li, 2007; Huang, 2011; Chen, 2013). On the other hand, *Treasury of Letters on Materia Medica*, published in 1655 (Gu, 2015), described that to treat *phlegm*-damps, PTM was better than raw PT. At the end of the 18th century and the early 19th century, several kinds of PTM products were developed for the treatment of different kinds of *phlegm*. Therefore, the objective for the processing of PT was not only to reduce the toxicities, but also to modify the efficacies for treating several kinds of *phlegm*.

In the successive Chinese Pharmacopoeias (Chinese Pharmacopoeia Commission, 2020), PT was first registered in the 1st edition, published in 1953, as the name of “Pinellia”. However, there were no descriptions about the processing. The 2nd edition, published in 1963, registered the item PT named as “Rhizoma Pinelliae,” and the sub-items of PPT, APT, and GPT as processed PT. The Chinese Pharmacopoeia 4th edition, published in 1985, newly registered PTM that was processed using APT with ginger juice, alumen, and rice malt in the appendix. In the Chinese Pharmacopoeia 2005 edition, PPT, APT, and GPT were separated as independent items from PT. PTM in the appendix was deleted in the Chinese Pharmacopoeia 2015 edition, and the style in the present Chinese Pharmacopoeia has been established (Chinese Pharmacopoeia Commission, 2020).

As far as the drug taste and property of PT were concerned, slightly cold (for raw PT) or warm (for processed PT) have been described in the literature published during the Eastern Han dynasty (Su, 2013; Tao, 2013), and pungent has been described in the Northern and Southern dynasties (Chen and Wu, 2013). The descriptions were then changed into bitter and pungent in the Yuan and Ming dynasties (Xu, 1384; Wang, 2008; Wang, 2011; Chen, 2013; Xue, 2015). Since the Qing dynasty, the drug taste and property have usually been described as pungent and warm (Yang, 1958; Huang, 1979; Zhang, 2011; Zhou, 2012; Wu, 2013; Wang, 2014), and the current Chinese Pharmacopoeia adopts these classifications (Chinese Pharmacopoeia Commission, 2020).

### Descriptions About PT Processing in the Successive Literatures Published in Japan

The descriptions are shown in Supplementary Table S3. The first description of PT in Japan was in *Honzo-wamyo*, published in ca. 918 (Fukane, ca. 918). However, there were no descriptions of the processing. The oldest processing method for PT was recorded in *Yurin-fukuden-ho*, published in 1363 (Yurin, 1363), describing processing using ginger.

In 1623, the fermented PT product, named PTM, was first recorded (Manase, 1623a; Manase, 1623b). For the preparation of PTM, powdered PT was mixed with ginger to make dumplings, and then, they were fermented for 2–3 days to become yellowish. After that, descriptions of PTM continually appeared from 1681 (Endo, 1681) to 1929 (Isshiki, 1929). In 1685, a new method to prepare PTM was developed, and the drug adjuvants used were ginger as well as alumen soup. In addition, fermentation was conducted by wrapping it with the leaves of *Broussonetia* sp. (Shimotsu, 1685). *Gleditschia japonica* hull, bamboo juice, *Vitex negundo* var. *cannabifolia* stem sap, white mustard powder, and reed sap first appeared for the preparation of PTM as drug adjuvants in 1698 (Okamoto, 1698; Ino, 1702). Several preparation methods using different drug adjuvants with or without using *Broussonetia* sp. leaves in the fermentation process have been developed since the end of the 17th century (Shimotsu, 1685; Okamoto, 1698; Ino, 1702; Katsuki, 1734; Kato, 1780; Terashima, 1824). The low-quality small-sized PT that could not be sold at drug stores seems to be used as the raw materials for PTM (Endo, 1681; Shibata, 1811; Ono, 1790). Shibata also described that PTM should be prepared by physicians on their own because the quality of PTM sold in drug stores is low which could be harmful (Shibata, 1811).

In 1685, the item name “PT cake” was first recorded in *Zukai-honzo* (Shimotsu, 1685). “PT cake” was simply prepared by mixing powdered PT with ginger juice without fermentation, and had sometimes appeared in the literature by 1824 (Terashima, 1824). In *Zukai-honzo*, the item name “PT powder” was also recorded, that was not the powdered PT but prepared by soaking powdered PT in water containing ginger juice and then dried (Shimotsu, 1685). The item “PT powder” had appeared in the literature published in 1698 (Okamoto, 1698), 1734 (Katsuki, 1734), and 1780 (Kato, 1780).

In 1738, Shuan Kagawa contradicted the toxicity of PT prepared as a decoction. He and his colleagues found that when the decoction of PT was prepared properly, the irritating taste at the throat disappeared, and he claimed that PT should not be processed (Kagawa, 1738). In 1771, Todo Yoshimasu agreed with Kagawa’s opinion, and claimed in his literature that the processing of PT with ginger killed the efficacy of PT and that PT should not be processed (Yoshimasu, 1771). This opinion was followed by Hisakata Naito in 1840 (Naito, 1840), and the descriptions about the processing of PT did not appear in *Yakuga*, published in 1848 (Taki, 1848), and *Koho-yakugi*, published in 1863 (Asada, 1863), although PT was recorded. Keisetsu Otsuka also opposed the description of the toxicities of PT in *Compendium of Materia Medica* by Shi-zhen Li (Li, 2004) in 1939 (Otsuka, 1939).

The purpose of the processing of PT was first described as the reduction of the “toxicity” of PT in 1581 (Manase, 1581), and the same intentions appeared in the literature published in 1681 (Endo, 1681), 1710 (Okunishi, 1710), 1811 (Shibata, 1811), 1824 (Terashima, 1824), and 1850 (Kitamura, 1850). This “toxicity” was speculated as causing throat irritation by Kagawa in 1738 (Kagawa, 1738) and by Naito in 1840 (Naito, 1840). However, there had been no other specific toxicological effects described in other literatures. In 1698, the efficacies of PTM were described in several ways, depending on the drug adjuvants (Okamoto, 1698). Similar descriptions appeared in 1702 (Ino, 1702) and in 1734 (Katsuki, 1734). In 1780, Kato described that PTM using ginger juice and alumen soup for not only removing the toxicity but also strengthening *spleen qi*, and described new processed PT product “Boiled PT (煮半夏)" and “PT prepared with four kinds of processing (四製半夏)".

The Japanese Pharmacopoeia has registered PT since the 5th edition, published in 1939, until the present 18th edition without any processing methods (The Society of Japanese Pharmacopoeia, 2021).

## Discussion

### History of the Preparation Methods of “Processing (熟)" for PT Before the Song Dynasty

In TCM medicinal theory, crude drugs have five kinds of tastes (pungent, sweet, bitter, sour, and salty) and properties (hot, warm, neutral, cool, and cold), and are classified based on these characteristics (Bensky et al., 2004). *Miscellaneous Records of Famous Physicians*, published during the Eastern Han dynasty, described that the drug property of PT was changed from slightly cold into warm by processing, and that the processed PT was the one washed with hot water to remove the viscous skin (Tao, 2013). In this literature, such differences in drug properties between raw and processed crude drugs appeared in only five crude drug items. Among them, the properties of PT, *Artemisia* sp. leaf, and arsenolite changed from cold to warm tendencies, while those of *Croton tiglium* seed and *Zanthoxylum bungeanum* pericarp changed from warm to cold upon processing. The processing of washing with boiling water was found in three crude drug items in this literature, but processing by washing with hot water appeared only in PT. In this era, *Collective Commentaries on Classics of Materia Medica* and *Classics of Golden Chamber and Jade Case* described that the operation of washing PT with hot water should be repeated for more than ten times to remove the viscous skin completely (Tao, 1997; Zhang, 2009). These descriptions revealed that repeated washing with hot water changed the drug properties of PT from slightly cold to warm.

Meanwhile, *Collective Commentaries on Classics of Materia Medica* described that the repeated washing with hot water could be substituted by boiling (Tao, 1997; Chen and Wu, 2013). Since *Synopsis of the Golden Chamber* also described that PT is used after boiling in water to remove the scum (Zhang, 2000), it is possible that the two different processing methods of PT, that is, repeated washing PT by water or boiling PT in water, co-existed before the Northern and Southern dynasties. *Supplement to Prescriptions Worth a Thousand Gold Pieces* published in the Tang dynasty described that the processing of washing and removing the viscous skin was necessary because PT caused irritation at the throat. In the Eastern Han dynasty, the objective of processing for PT was to change the drug properties and to detoxify irritation at the throat when orally administered according to the description in the *Classics of Golden Chamber and Jade Case* (Zhang, 2009). Subsequently, the item name of processed PT had been appeared in the later literature.

The processing of PT using ginger first appeared in the *Handbook of Prescriptions for Emergency* published in the Eastern Jin dynasty (Ge, 2016), although the objective of ginger was not described. In Northern and Southern dynasties, *Collective Commentaries on Classics of Materia Medica* described that it was necessary to add ginger in formulas to detoxify PT. Although the processing of PT using ginger and the combination of PT and ginger in a formula are different processes to prepare the drug products, the objective of ginger for the processing of PT would be to promote the detoxification of PT by simply washing with hot water. Indeed, processing with ginger causes structural changes in the raphides and reduces irritating activity at the throat (Wu et al., 2010; Fueki et al., 2020). Since the Tang dynasty, the processing of PT using ginger has become common to detoxify irritation at the throat (Sun, 1982), and in the Song dynasty, the processing of PT was further developed from simply washing with hot water containing ginger to mashing PT with ginger to make PT cake and PTM (Taiping People’s Welfare Bureau, 1975).

### Transition of the Drug Taste and Drug Property of PT

The first descriptions of the drug taste and the properties of PT were pungent and neutral in *Shennong’s Classic of Materia Medica*, published in the Eastern Han dynasty (Wang, 2010), and *Miscellaneous Records of Famous Physicians* described them as slightly cold (raw) or warm (processed) (Tao, 2013). Since then, these classifications have appeared in the successive medicinal literature in mainland China until the 16th century, and subsequently changed by *Origins of Materia Medica,* published in 1612 (Li, 2007). *Orthodox Materia Medica*, published in 1624 (Zhang, 1624), first described the drug property of unprocessed PT as warm, while the description of the drug taste was pungent as before. Identical descriptions of the drug taste and the properties of PT as pungent and warm have been described in the literature following *Orthodox Materia Medica* up to the current Chinese Pharmacopoeia. This alteration in the drug properties of PT that occurred around the early 17th century in mainland China could be related to the alteration in the usage of PT. The drug properties of processed PT have been described as warm before this alteration. As unprocessed PT was used less and less, and the processed PT became popular, the processed PT may have become the standard in this era and the alteration of drug properties may have followed this stream. In the medical literature, this theory is supported by the fact that unprocessed PT was used alone until the Jin and Yuan dynasties. However, it was generally replaced by processed PT products after the Ming dynasty, and in rare cases of unprocessed products, they were used together with ginger. Indeed, the current Chinese Pharmacopoeia describes that PT as toxic, and oral use only after being processed in general (Chinese Pharmacopoeia Commission, 2020).

This alteration in the drug properties of PT was transported into Japan. The earlier classification of pungent and slightly cold (raw) or warm (processed) in PT was found in Japanese literature from 1623 to 1824, and the latter classification of pungent and warm was seen from 1772 to the latter 20th century. In Japanese Kampo medicine, the processing of PT has not been popular since the 18th century, and because the theory about the efficacy of a single crude drug has not been well developed compared to the theory about the usage of formulas (Yasui, 2021; Makino et al., 2022). This alteration of drug property of PT in mainland China might not have affected the medicinal theory in Kampo medicine.

### Transition of the Meaning of PTM

The origin of PTM has been changed over a long history. The term “PTM,” without a description of the preparation method, was first found in *Huatuo’s devine formulations* (Hua, 2010). However, there have been discussions regarding the establishment of this literature. For example, Si-miao Sun described in the preface of this reprinted book that the assertion that this literature had been published before Tang dynasty was questionable because this literature contains references from *Formulary of Universal Relief* published in the Ming dynasty, and because the usage of the term “qian” as a weight unit was present in the literature, although this unit was established long after the Tang dynasty (Sun, 2010).

The first description of the preparation method for PTM was in the *Formulary of the Bureau of Taiping People’s Welfare Pharmacy*, published in 1078–1085 (Taiping People’s Welfare Bureau, 1975). In this literature, PTM was prepared by mixing mashed PT with ginger juice and alumen on the agenda of “Cinnabar-phlegm-resolving pill” formula. *Comprehensive Recording of Sage-like Benefit*, published in 1117 (North Song Imperial Government, 1962), described PTM as a mixture of PT and ginger. *One Hundred Questions about Woman’s Diseases*, published in 1220 (Qi, 2012), described PTM as a mixture of powdered PT and ginger juice with mild boiling and the taste being not spicy. In summary, PTM was prepared using PT and ginger, with or without alumen, and without any other drug adjuvants during the Song dynasty.

The history of PTM after the Song dynasty differs between the medical and medicinal literature. In the medical literature, the preparation method is listed in the *Formulary of the Bureau of Taiping People’s Welfare Pharmacy*, as shown above. In the Ming dynasty, the PPT appeared in the *Restoration of Health from the Myriad Diseases* (Gong, 2013). In the Qing dynasty, the number of formulas prescribed for PTM decreased, and PTM was replaced by PPT. In medicinal literature published in the Ming dynasty, the name of PTM was first appeared in *Compendium of Materia Medica* (Li, 2004), with a description of its efficacy in removing *phlegm*. In the Qing dynasty, PPT first appeared in the *Supplement to Compendium of Materia Medica* (Zhao, 1983), and its contents were derived from the description in the *Restoration of Health from the Myriad Diseases* (Gong, 2013)*.* However, PTM was listed in this literature.

As discussed in detail, the number of drug adjuvants widely increased during the Ming dynasty, and fermentation was introduced to prepare PTM. *Compendium of Materia Medica*, published in 1578 (Li, 2004), defined PTM as the product derived from dumplings made from powdered PT treated with several kinds of drug adjuvants such as ginger juice, alumen, *Gleditschia japonica* hull soup, bamboo sap, *Vitex negundo* var. *cannabifolia* stem sap, or white mustard powder, and fermented in the leaves of *Broussonetia* sp. Drug adjuvants were chosen according to the types of *phlegm* to be removed. These descriptions were reflected by *Han’s General Medicine*, published in 1522 (Han, 1522). On the other hand, other literatures published in the Ming dynasty compared to the *Compendium of Materia Medica* (Li, 2004) recorded each type of PTM, that is, PTM using both ginger and alumen (Chen, 2013) and three drug additives of ginger, alumen and *Gleditschia japonica* hull (Li, 2015). Furthermore, the processed PT product simply treated with alumen was referred to PPT in *Restoration of Health from the Myriad Diseases*, published in 1587, and “PPT” as the item name of processed PT product was first used in this literature. It was observed that the processing of PT had been highly developed and that the meaning of the term PTM had been changed and confused in the Song dynasty through the Ming dynasty. As a result, the origin of PTM varied depending on the literature.

The variation in the substances indicated by the term PTM indicated was transported into Japan. The first description of PTM in Japanese literature was *Wamyoshu-narabini-imyo-seizaiki*, published in 1623 (Manase, 1623b), describing that PTM is a fermented PT product treated only with ginger. Identical descriptions were found in *Yamato-honzo*, published in 1709 (Kaibara, 1709), and *Yakushu-shin-seizaiki* in 1710 (Okunishi, 1710). On the other hand, *Koeki-honzo-taisei*, published in 1698 (Okamoto, 1698), and *Hosha-zensho*, published in 1702 (Ino, 1702), described the multiple PTM products treated with several kinds of drug adjuvants and fermented according to *Compendium of Materia Medica* (Li, 2004), which was followed by *Yakuro-honzo*, published in 1734 (Katsuki, 1734). Therefore, the literature published in Japan in the 17th and 18th centuries is reflected in the literature published during the Ming dynasty.

In the Qing dynasty, drug adjuvants to prepare PTM and PPT were developed. For the preparation of PPT, ginger, alumen, lime, children’s stool, mirabilite, radish juice, bamboo sap, the decoction of the mixture of licorice root, peppermint leaves, *Trichosanthes kirilowii* rhizomes, immature orange fruits, citrus peels, *Amomum villosum* seeds, and *Saussurea costus* roots were used as drug adjuvants (Tao, 1759). It is also found that the multiple PTM products had come to have the individual names such as “ginger malt,” “alumen malt,” etc., and new drug adjuvants also had been developed using the substances derived from not only plants and minerals but animals (Huang, 1979).

However, the development of drug adjuvants used for the preparation of PTMs in the Qing dynasty did not seem to have been transported into Japan. Moreover, the definition of PTM reverted to a simple one in Japan. *Hengyoku-rokuhachi-honzo*, published in 1780 (Kato, 1780), and *Wakan-sansai-zue*, published in 1824 (Terashima, 1824), defined PTM as a fermented PT product treated with ginger juice and alumen soup, and *Wakanyaku-no-ryohi-kanbetsuho-oyobi-choseiho*, published in 1929 (Isshiki, 1929), described that PTM was the fermented PT product treated with ginger. These descriptions are similar to those of the Song dynasty in mainland China.

In the latter 20th century, PTM appeared in the Chinese Pharmacopoeia from 1985 to 2005 in the appendix, indicating that the PT processed using APT with ginger juice, alumen, and rice malt, had disappeared from the 2015 edition (Chinese Pharmacopoeia Commission, 2020). In China, the term PTM refers to multiple fermented products containing PT with several independent names, similarly to the situation in the Qing dynasty. The common efficacy of multiple PTM products is to remove *phlegm* and to promote digestion, and the variation of each PTM product is very large depending on the drug adjuvants (Shen et al., 2009; Zhang et al., 2015b). Because the current PTM was not a single crude drug, it would be very difficult to make the standard for PTM, and then, Chinese Pharmacopoeia deleted PTM as the drug item.

As described above, the meaning of the term “PTM” has been changed repeatedly, and when we discuss something using the term “PTM,” the definition of “PTM” is needed.

### Processed PT Described in the Formulary of the Bureau of Taiping People’s Welfare Pharmacy

The *Formulary of the Bureau of Taiping People’s Welfare Pharmacy* listed three agendas of “New-prepared*-*PT decoction formula” with the same item name, while the contents of each formula are different. Interestingly, the types of processed PT used in each formula were also different. That is, the 1st one was washed PT with ginger juice, the 2nd one contained PTM (roasted), and the 3rd one contained “Big PT” prepared by the description in Supplementary Table S1. The efficacies of these three decoctions were to treat *spleen-stomach* disharmony syndrome and to remove *phleural* fluid retention in common, while other minor symptoms were different. Since these efficacies were not obtained by single PT products, the differences in the efficacies of each processed PT product could not be evaluated. However, these might depend on the different types of *phleural* fluid syndromes or their severities according to the descriptions in the subsequent literature. The name of the formula “New-prepared*-*PT decoction” contains the phrase “new-prepared-PT”. However, this phrase would not be the meaning of “new PPT,” which is defined in the current Chinese Pharmacopoeia, because it is considered that the target of the adjective phrase “new-prepared” was not “PT” but “PT decoction”. Since the prior literature before the *Formulary of the Bureau of Taiping People’s Welfare Pharmacy* had never described “prepared PT,” there was no target of the adjective “new” in the formula name at this era, and *Arcane Essentials from the Imperial Library*, published in 752 (Wang, 752), had already described “PT decoction".

### Why the Current Kampo Medicine Does Not Process PT?

The earliest literature describing the processing of PT in Japan was *Yurin-Fukuden-ho* published in 1363, and the processing of PT was instructed strictly in order both to reduce the toxicities and to change the drug properties in the subsequent literature published in Japan. These descriptions may have been highly affected by descriptions in the medical and medicinal literature published in mainland China. Indeed, the descriptions of the processing of PT in *Koeki-honzo-taisei* (Okamoto, 1698), *Hosha-zensho* (Ino, 1702), *Yakuro-honzo* (Katsuki, 1734), *Hengyoku-rokuhachi-honzo* (Kato, 1780), *Nichiyo-yakuhin-ko* (Shibata, 1811), and *Wakan-sansai-zue* (Terashima, 1824) were almost the same as those in *Compendium of Materia Medica* (Li, 2004). Indeed, the descriptions of “PT cake” and “PT powder” also appeared in these literatures.

On the other hand, critical descriptions of PT processing first appeared in *Ippondo-yakusen* (Kagawa, 1738). According to the description in this literature, the author Shuan Kagawa and his colleagues actually drank the decoction of PT by themselves, and most of them did not find any acrid pain at the throat. Based on this practical finding, he concluded that PT should not be processed because the toxicity of PT would disappear by proper preparation of the decoction. Indeed, the irritant taste of PT disappears when PT is decocted in boiling water for approximately 30 min in our previous study (Fueki et al., 2016). *Ippondo-yakusen* also estimated the effectiveness of the processing of the root of *Angelica acutiloba* and the rhizome of *Rheum palmatum* negatively (Kagawa, 1738). This policy might have influenced Japanese Kampo medicine in which these two crude drugs were also not generally processed, while the root of *Angelica sinensis* and the root and rhizome of *Rheum palmatum* are commonly processed in TCM (Chinese Pharmacopoeia Commission, 2020).

Todo Yoshimasu followed Kagawa’s experiences, and described in his literature (Yoshimasu, 1771) that the processing of PT with ginger killed the efficacy of PT. Therefore, he said that PT should not be processed. Since Yoshimasu was a materialist and a positivist, and he believed only visible things and denied the theory of *Yin-Yang* and the five elements, he regarded *Treatise on Cold Damage* published by Zhong-jing Zhang in the Eastern Han dynasty (Zhang, 2000) as the best medical textbook, and established the basic theory of Japanese traditional Kampo medicine (Daidoji, 2013). Their opinion was followed by *Koho-yakuhin-ko*, published in 1840 (Naito, 1840).

Finally, Keisetsu Otsuka, who restored and modernized Japanese traditional Kampo medicine (Otsuka, 2016), and highly influenced the community of Kampo medicine as one of the founders of the Japanese Society of Oriental Medicine (Yasui, 2021), contradicted the description of the toxicities of PT in the *Compendium of Materia Medica* by Shi-zhen Li (Li, 2004), and described that the processing of PT using ginger was not needed because it killed the efficacy of PT in 1939 (Otsuka, 1939).

In the literature published in mainland China, the critical descriptions for the processing of PT were seen in the *Supplement to Compendium of Materia Medica* published in 1765, *Orthodox Interpretation of Materia Medica* in 1828, and *Harm and Benefit in Materia Medica* in 1862. However, these critical opinions have been ignored, and the current Chinese Pharmacopoeia recommends the use of processed PT to avoid the toxicity. Positivism has been one of the characteristics of Kampo medicine (Yasui, 2021), and this policy has also reflected the differences in the processing of PT between Kampo medicine and TCM.

## Conclusion

In the long history of mainland China from the Han dynasty to modern era, the methods and objectives of the processing of PT had changed repeatedly, and the current processing methods have been established in the Qing dynasty. The objective of the processing for PT was not only to remove its toxicity but to change drug properties, and several kinds of processed PT had been developed to treat different types of *phlegm* in the Ming dynasty. Transition of the drug taste and drug property of PT had also changed both in China and Japan, but the alteration of the drug property of PT in mainland China had not affected the medicinal theory in Kampo medicine. The current Chinese Pharmacopoeia recommends the use of processed PT to avoid the toxicity, while PT is not processed in Japan. Since the toxicity of PT about causing throat irritation is actually disappeared by preparing its decoction, Japanese traditional Kampo medicine has not employed the processing for PT to avoid the loss of its effectiveness since 18th century. Compared to TCM, Japanese Kampo medicine has tended to avoid ideologism based on traditional knowledge and to adopt positivism. This policy has reflected the differences in the processing of PT between Kampo medicine and TCM.

## Data Availability

The original contributions presented in the study are included in the article/Supplementary Material, further inquiries can be directed to the corresponding author.
