# Supplementary material for: Historical Study for the Differences of Processing of Pinellia ternata Tuber Between China and Japan
Source: Front Pharmacol. 2022 Jun 20;13:892732. doi: 10.3389/fphar.2022.892732 (PMC9251410; doi:10.3389/fphar.2022.892732)
Supplement: Supplementary file 1 [file Table1.pdf]

Supplementary Table 1. The descriptions about processing of Pinellia Tuber (PT) in the medical literatures published in mainland China.

Year	Author	Literature	Descriptions about PT processing
Eastern Han	Zhong-jing Zhang (The Japan Kampo Medicine Association, 2000)	*Treatise on Cold Damage* (傷寒論)	Use PT after washing. PT has toxicity and the oral administration as the powdered form is not suitable.
Eastern Han	Zhong-jing Zhang (The Japan Kampo Medicine Association, 2000)	*Synopsis of the Golden Chamber* (金匱要略)	Use PT after boiling in water and removing the scum.
Eastern Han	Zhong-jing Zhang (Zhang, 2009)	*Classics of Golden Chamber and Jade Case* (金匱玉函經)	Wash PT without cutting using hot water ten or more times, and then wash them using cold water to remove the viscous skin completely. If the washing is not enough, they are toxic.
Eastern Han?	Tuo Hua (Hua, 2010)	*Huatuo's devine formulations* (華佗神方)	*Qitanshenfang*: *Arisaema heterophyllum* rhizome malt, PT malt (半夏麹, PTM), and citrus peel. Mash these three kinds of drugs in ginger juice to make pills with the size of *Firmiana simplex* fruit.
Eastern Jin	Hong Ge (Ge, 2016)	*Handbook of Prescriptions for Emergency* (肘後備急方)	Soak PT in the decoction of *Physalis alkekengi* for one day, wash it in hot water five–seven times to remove evil *qi*, dry them under sunlight, mash them into powder, add water to make cakes, dry them under sunlight, and mash them into powder again to make pills.<br>PT should be washed with hot water five–six times.<br>Wash-processing (熟洗) PT and take them as powdered form.<br>Wash-processing (熟洗) PT, dry and powder them, and take it with ginger and hot water.

442 Northern and Southern dynasties	Juan-zi Liu (Liu, 1970)	*Liu Juanzi's Ghost-Bequeathed Prescriptions* (劉涓子鬼遺方)	Wash PT with hot water seven times, soak them with ginger for one night, and boil them.
682 Tang	Si-miao Sun (Sun, 1982)	*Supplement to Prescriptions Worth a Thousand Gold Pieces* (千金翼方)	Since PT cause the irritation at throat, the processing is necessary. Use them after washing and removing the viscous skin. Do not take the juice. It is better to add ginger.<br>Roast PT under mild heating, mash them into powder, and mix with liquor to make pills with the size of foxtail millet.
1078-1085 Song	Taiping People's Welfare Bureau (Taiping People's Welfare Bureau, 1975)	*Formulary of the Bureau of Taiping People's Welfare Pharmacy* (太平惠民和劑局方)	Processed PT (熟半夏)<br>Soak PT in boiling water to remove the viscous skin. Repeat this process seven times for using PT as the decoction pieces. If PT still cause the irritation at throat, powder PT using beetle, mash it with ginger, submerge them one night, make cakes, then roast and dry them.<br>Wash PT seven times, mash them with ginger juice and mix alumen to prepare PTM.<br>New-prepared-PT decoction formula: Wash PT with hot water containing ginger juice.<br>Cut PT into four slices, soak them in ginger juice for one night, and then stir-fry them with foxtail millet until yellowish.<br>New-prepared-PT decoction formula: PTM roasted.<br>New-prepared-PT decoction formula: Big PT (大半夏): Wash PT with hot water seven times, cut into slices, soak them in boiled water containing alumen and keep one night, wash alumen by hot water, and dry them. Soak them again in ginger juice for one night, dry them using mild fire, and be powdered. Make dumplings using ginger juice, dry and roast them into yellowish and dark brown.
1117 Song	North Song Imperial Government (North Song Imperial Government, 1962)	*Comprehensive Recording of Sage-like Benefit* (聖濟總錄)	Mix powdered PT with ginger to make PTM.<br>Grind PT into powder, mash them in ginger juice to make dumplings, roast to dry them, and then make powder again.<br>Stir-fry PT with *Polyporus umbellatus* sclerotial into purple color.<br>Boil PT mildly in water with licorice root and *Morus alba* root cortex.<br>Stir-fry PT with rice into slightly yellowish.<br>Stir-fry PT with foxtail millet and rice into yellowish, and then remove rice.

			Soak PT in water containing alumen, then roast and dry them.<br>Soak PT in vinegar for one day, boil and bake them.
1146<br>Song	Cai Dou<br>(Dou, 2016)	*Bianque's Heart Book*<br>(扁鵲心書)	Stir-fry PT in the decoction of ginger, alumen, and *Gleditsia sinensis* hulls.
1220<br>Song	Zhong-fu Qi<br>(Qi, 2012)	*One Hundred Questions about Woman's Diseases*<br>(女科百問)	Wash PT seven times, mash them into powder in ginger juice for seven or eight times until their taste became not spicy to prepare PTM.
1347<br>Yuan	Zhen-heng Zhu<br>(Zhu, 1347)	*Danxi's Experiential Therapy*<br>(丹溪心法)	Soak PT in the water containing *Gleditsia sinensis* hulls and dry them.<br>Soak PT in the slop from rinsing rice for one day.<br>Chop PT, and stir-fry them with sesame oil.
1522<br>Ming	Mao Han<br>(Han, 1522)	*Han's General Medicine*<br>(韓氏醫通)	Since PT is pungent, making malt is necessary. Mash PT in ginger juice or alumen soup to make dumplings, wrap them with the leaves of *Broussonetia* sp., ferment and dry them.<br>For *wind phlegm*, PT is prepared with the decoction of *Gleditsia sinensis* hulls.<br>For *fire phlegm*, PT is prepared with ginger juice containing bamboo sap or *Vitex negundo* var. *cannabifolia* stem juice.<br>For *moist phlegm* and white *cold phlegm*, PT is prepared using concentrated ginger decoction with alumen before making PTM.
1565<br>Ming	Ying Lou<br>(Lou, 1565)	*Compendium of Medicine* (醫學綱目)	Mash PT with ginger, and then roast them until yellowish.<br>Boil PT in the water containing ginger juice.<br>Soak one part of PT in alumen soup. Soak the second part of PT in the water containing powdered *Gleditsia sinensis* hulls. Boil the third part of PT in the water containing *Croton tiglium* seeds. After ten days, remove *Gleditsia sinensis* hulls and *Croton tiglium* seeds, mix and boil them mildly to dry.<br>Wash PT with liquor, and roast them.
1587<br>Ming	Ting-xian Gong<br>(Gong, 2013)	*Restoration of Health from the Myriad Diseases*<br>(萬病回春)	Prepared PT (PPT): For removing *phlegm* like Gods or unbelievable effects, wash with clean water. For the diseases of *phlegm* with stroke and reticence, wash PT with water containing flowers. By scuffing the abdomen with them using hands, soon wake up and open the mouth. Soak big size PT in cold water containing several drug adjuvants including lime, alumen, and mirabilite in a sequential manner. After dried under sunlight, wash them using clean water for three–four times, soak in water for three days with daily exchange, and dry them.

1640<br>Ming	Jie-bin, Zhang<br>(Zhang, 2015)	*Jing Yue's Collected Works* (景岳全書)	Since the administration of raw PT cause the irritation at throat, process PT with ginger.
1759<br>Qing	Tao Cheng-xi<br>(Tao, 1759)	*Huizhitangjingyanfang* (惠直堂經驗方)	Prepared PT (PPT): Soak big size PT in hot water containing drug adjuvants in a sequential manner as follows: lime; children's stool; mirabilite and alumen; ginger decoction; radish juice; bamboo sap; the decoction of the mixture of licorice root, peppermint leaves, *Scutellia baicalensis* roots, *Trichosanthes kirilowii* rhizomes, immature orange fruits, citrus peels, *Amomum villosum* seeds, and *Saussurea costus* roots. Then, heat and dry them.<br>PTM: Mash PT with ginger and flour in the juice of *Persicaria* sp. aerial parts and the decoction of *Gleditsia sinensis* hulls, make dumplings, and ferment them in a basket for seven days. Dry them by wind.

References:

Dou, C. (2016). Rpt. *Bianque's Heart Book* (扁鹊心书), Tokyo: Digital Collections of Keio University Libraries. p. 14. https://dcollections.lib.keio.ac.jp/ja/koisho/f-he-10-2

Ge, H. (2016). Rpt. *Handbook of Prescriptions for Emergency* (肘後備急方), in: Proofreading and annotation of *Handbook of Prescriptions for Emergency,* Ed. S.N. Shen. Beijing: People's Health Publisher. p. 10, 68, 73, 149.

Gong, T.X. (2013). Rpt. *Restoration of Health from the Myriad Diseases* (万病回春), Ed. T. Sou. Beijing: People's Medicinal Publishing House. p. 117.

Han, M. (1522). *Han's General Medicine* (韩氏医通), Kyoto: Kyoto University Rare Material Digital Archive. p. 40. https://rmda.kulib.kyoto-u.ac.jp/item/rb00001738

Hua, T. (2010). Rpt. *Huatuo's devine formulations* (华佗神方), in: Huatuo's devine medicinal esotericism, Eds. J.S. Peng & C.Y. Wang. Shenyang: Liaoning Science and Technology Publishing House. p. 116.

Liu, J.Z. (1970). Rpt. *Liu Juanzi's Ghost-Bequeathed Prescriptions* (刘涓子鬼遗方), in: Liu Juanzi's Ghost-Bequeathed Prescriptions and Essence of External Diseases, Ed. Taiyuan: Shanxi Science & Technology Press. p. 13.

Lou, Y. (1565). Compendium of Medicine (医学綱目), Tokyo: National Archives of Japan. p. 23. https://www.digital.archives.go.jp/img/4109547

North Song Imperial Government (1962). Rpt. *Comprehensive Recording of Sage-like Benefit* (圣济总录), Ed. J. Zhao. Beijing: People's Medical Publishing House. p. 279, 470, 585, 798, 813, 2115, 2137.

Qi, Z.F. (2012). Rpt. *One Hundred Questions about Woman's Diseases* (女科百问), Beijing: China Medical Science Press. p. 70.

Sun, S.M. (1982). Rpt. *Supplement to Prescriptions Worth a Thousand Gold Pieces* (千金翼方), Taipei: Hongye Publishing. p. 137.

Taiping People's Welfare Bureau (1975). Rpt. *Formulary of the Bureau of Taiping People's Welfare Pharmacy* (太平惠民和剂局方), Ed. S.W. Chen. New Taipei City: Xuanfeng Publishing. p. https://dl.ndl.go.jp/info:ndljp/pid/2555873

Tao, C.X. (1759). Rpt. *Huizhitangjingyanfang* (惠直堂經驗方), in: *Zhenbenyishujicheng* (珍本醫書集成), Ed. J.S. Qiu. Taipei: National Central Library. p. 231. https://taiwanebook.ncl.edu.tw/zh-tw/book/NTUL-0521059

The_Japan_Kampo_Medicine_Association, (2000) Rpt. *Treatuse on Cold Damage and Synopsis of thre Golden Chamber,* Toyo Gakijutsu Shuppansha, Tokyo, p. 139, 312.

Zhang J.Y, 2015. Rpt: *Jing Yue's Collected Works* (景岳全書), Taiyuan: Shanxi Science and Technology Publishing House. pp. 631.

Zhang, Z.J. (2009). Rpt: *Classics of Golden Chamber and Jade Case* (金匮玉函经), in: Study of *Classics of Golden Chamber and Jade Case* (金匮玉函经研究), Ed. Z.W. Wu. Beijing: TCM Classics Publishing House. p. 297.

Zhu, Z.Z. (1347). *Danxi's Experiential Therapy* (丹溪心法), Tokyo: National Archives of Japan. vol. 1, 64; vol. 62, p. 51. https://www.digital.archives.go.jp/file/1084008
