# Supplementary material for: Historical Study for the Differences of Processing of Pinellia ternata Tuber Between China and Japan
Source: Front Pharmacol. 2022 Jun 20;13:892732. doi: 10.3389/fphar.2022.892732 (PMC9251410; doi:10.3389/fphar.2022.892732)
Supplement: Supplementary file 2 [file Table2.pdf]

Supplementary Table 2. The descriptions about processing of Pinellia Tuber (PT) in the medicinal literatures published in mainland China.

Year	Author	Literature	Taste and Property	Descriptions about PT processing
Eastern Han	Unknown (Tao, 2013)	*Miscellaneous Records of Famous Physicians* (名醫別錄)	Slightly cold (raw). Warm (processed).	Raw PT cause vomiting, processed PT cause diarrhea. Use PT after washing with hot water to remove the viscous skin.
ca. 536 Northern and Southern dynasties	Hong-jing Tao (Tao, 1997; Chen and Wu, 2013)	*Collective Commentaries on Classics of Materia Medica* (本草經集注)	Pungent. Neutral. Slightly cold (raw). Warm (processed).	Wash PT using hot water to remove its skin by hand, and then wash again using hot water to remove its viscous skin. If this process is not enough, PT cause the irritation at throat. Formerly, this process had been repeated twenty or more times, but now repeated six or seven times and it is enough. Alternatively, directly boiling PT in water is easy, and repeating three times is enough and washing should be stopped. Cut PT into small pieces, and use them to prepare decoctions. For ointment, liquor, pill, or powdered prescriptions, dry them completely.<br>Wash PT ten or more times using hot water to remove its s viscous skin. For the formula containing ginger, ginger is necessary since PT is toxic.
588 Sui	Xiao Lei (Lei, 2010)	*Master Lei's Discourse on Medicinal Processing* (雷公炮製藥性解)	No records.	Mash PT with the white mustard and vinegar and wash them three times.
ca. 600 Tang	Quan Zhen (Zhen, 1983)	*Treatise on Medicinal Properties* (藥性論)	No records.	Wash PT viscous skin with hot water, and remove the toxicities by the processing using ginger.
659 Tang	Jing Su (Su, 2013)	*Newly Revised Materia Medica* (新修本草)	Pungent. Neutral. Slightly cold (raw). Warm (Processed).	Wash PT viscous skin with hot water. Since PT is toxic, processing with ginger is necessary. The processing using ginger removes the toxicities.<br>(The same expressions described in *Collective Commentaries on Classics of Materia Medica* mentioned above were appeared again.)

1116 Song	North Song Imperial Government (Chen and Wu, 2013)	*Revised Zhenghe Classified Materia Medica from Historical Classics for Emergency* (重修政和經史證類備用本草)	Pungent. Neutral. Slightly cold (raw). Warm (Processed).	Since PT is toxic, processing with ginger is necessary.<br>Mash PT with white mustard in vinegar and wash them three times.
1248 Yuan	Hao-gu Wang (Wang, 2008)	*Materia Medica for Decoctions* (湯液本草)	Pungent. Slightly cold (raw). Warm (Processed).	Raw PT is used for vomiting. Processed PT is used for diarrhea.<br>Processed PT with ginger in order to remove *phlegm* and drool, to open *stomach*, and to strengthen *spleen*.
1520 Ming	Ji Xue (Xue, 2015)	*Bencaoyueyan* (本草約言)	Pungent and slightly bitter. Slightly cold (raw). Warm (Processed).	For pregnant women, the processing by stir-frying PT with ginger is necessary.<br>Alternatively, boil PT with the water containing ginger, alumen, and licorice root.
1552–1578 Ming	Shi-zhen Li (Li, 2004)	*Compendium of Materia Medica* (本草綱目)	Pungent. Neutral.	Soak PT in hot water with daily replacement of water for seven days. Dry and cut them, and roast them with ginger juice to prepare the drug.<br>Alternatively, grind PT, and soak them in hot water with ginger juice for three days. After removal of the sliminess in the supernatant, collect the deposit, and dry it to prepare "PT powder (半夏粉)". Alternatively, grind PT into powder, and mix it with ginger juice to make the cake. Then, dry under sunlight to make "PT cake (半夏餅)". Alternatively, grind PT into powder, mix it with ginger juice and alumen soup to make dumplings, wrap them with the leaves of *Broussonetia* sp., and ferment them. When yellowish coats are appeared, collect and dry them under sunlight to prepare PTM (半夏麯).

				According to Han's General Medicine (Han, 1522), in order to treat *phlegm*-damps, mix PT with ginger juice and alumen soup. To treat *wind phlegm*, mix them with *Gleditschia japonica* hull soup and ginger juice. To treat *fire phlegm*, mix them with with ginger juice, bamboo sap, or *Vitex negundo* var. *cannabifolia* stem sap. To treat cold *phlegm*, use ginger juice, alumen soup and white mustard powder to make malt.<br>Wash big size PT with hot water seven times, roast them, dry, wash them again, soak them in the slop from rinsing rice for one day, soak them in alumen soup for five days, roast and dry them, soak them in warm water containing white lead frost for seven days, boil them mildly, roast and dry again.<br>Wash PT in hot water seven times, make powdered PT, and soak it in water for three days. Filtrate them using silk to remove the waste, and dry them.
1560 Ming	Jia-mo Chen (Chen, 2013)	*Enlightneing Primer of Materia Medica* (本草蒙筌)	Pungent and slightly bitter. Neutral. Slightly cold (raw). Warm (Processed).	Mash PT with alumen and ginger juice to make dumplings, wrap them in the leaves of *Broussonetia* sp., and ferment them to make PTM.<br>The efficiencies of raw PT are sharp, and those of PTM are soft.
1578 Ming	Fu-song Huang (Huang, 2011)	*Illumination of Materia Medica* (本草發明)	Pungent. Neutral. Slightly cold (raw). Warm (Processed).	The efficiencies of raw PT are sharp, and those of PTM are soft.
1612 Ming	Zhong-li Li (Li, 2007)	*Origins of Magteria Medica* (本草原始)	Pungent. Neutral.	Soak PT in hot water with daily replacement of water for 2–3 days. Then, add *Gleditschia japonica* hull, alumen, and ginger juice to boil. Wait for cooling, wash with clean water, cut, and dry to use.<br>Raw PT cause throat irritation. Grind PT into powder, mix it with ginger juice and alumen soup to

				make dumplings, wrap them with the leaves of *Broussonetia* sp., and ferment them. When yellowish coats are appeared, collect and dry them under sunlight to prepare PTM.
1624 Ming	Jie-bin Zhang (Zhang, 1624)	*Orthodox Materia Medica* (本草正)	Strongly pungent and slightly bitter. Warm.	Biting raw PT cause throat irritation. Therefore, the processing using ginger is necessary.
1655 Ming	Zhong-zi Li (Li, 2015)	*Penetrating the Mysteries of Materia Medica* (本草通玄)	Pungent. Warm.	Soak big size white PT in water for seven days, remove skin, boil them in the water containing ginger juice, alumen, and *Gleditschia japonica* hull, dry them to make dumplings, wrap them with the leaves of *Broussonetia* sp., ferment them to become yellowish, and dry after the removal of leaves.
1666 Ming	Yuan-jiao Gu (Gu, 2015)	*Treasury of Letters on Materia Medica* (本草匯箋)	Pungent. Warm.	To treat *phlegm*-damps, using PT is good, and PTM is the best.
1695 Qing	Lu Zhang (Zhang, 2011)	*Encountering with Origin of Herbal Classic* (本經逢原)	Pungent. Warm.	Soak PT in the water containing *Gleditschia japonica* hull, boil them with alumen and ginger juice, and dry them.<br>For *cool-phlegm* in children or for the deficiency of *gallbladder* with not well sleeping, stir-fried PT with pig bile should be used.<br>The drug adjuvants of *Gleditschia japonica* hull, alumen, ginger juice, and bamboo sap are the best.<br>For throat pain, stir-flied PT with vinegar should be used.
1765 Qing	Xue-min Zhao (Zhao, 1983)	*Supplement to Compendium of Materia Medica* (本草綱目拾遺)	No records.	"Mountain Hermit's PT (仙半夏)", several kinds of PTM: Recently, physicians have used several kinds of PTM, and the drug stores have produced them. The preparation method has descended in Mountain Hermits, since this item name was used. Its effectiveness to remove *phlegm* is like Gods.<br>Soak big size PT in hot water containing drug adjuvants in a sequential manner as follows: lime; mirabilite and alumen; the decoction of the mixture of licorice root, peppermint leaves, cloves, cardamons, agarwoods, immature orange fruits, *Saussurea costus* roots, *Ligusticum chuanxiong* rhizomes, *Cinnamomum cassia* barks, citrus peels, *Schisandra chinensis* fruits, and *Amomum villosum* seeds; wrap them with cloths and heat them.<br>The preparation method of "Prepared PT (PPT)" was not as same as that of "Mountain hermit's PT".

				Recent drug stores treat "Mountain Hermit's PT" products using licorice root, but they are sweet and lose their drug properties.
1769 Qing	Gong-xin Huang (Huang, 1979)	*Seeking Accuracy in Materia Medica* (本草求真)	Pungent. Warm.	Boil PT with *Gleditschia japonica* hull, alumen, or ginger juice.<br>PT processed with *Gleditschia japonica* hull can treat *wind-phlegm*. PT processed with lime can treat *spleen and stomach-phlegm*. PT processed with alumen can treat *water-phlegm*. PT processed with ginger can treat *cold-phlegm*. PT processed with licorice root can treat the toxicities.<br>PT processed with ginger, alumen, *Gleditschia japonica* hull, bamboo sap, sesame oil, and cow's gallbladder are called "ginger malt", "alumen malt", "*Gleditschia japonica* malt", "bamboo sap malt", "sesame oil malt", and "cow's gallbladder malt", respectively. PT processed with *Cyperus rotundus* rhizome, *Atractylodes lancea* rhizome, *Ligusticum sinense* rhizome is called "opening depression malt". PT processed with mirabilite and rhubarb is called " mirabilite-rhubarb malt". PT processed with *Notarchus leachii* subsp. *cirrosus* eggs and realgar is called "*Notarchus leachii*-malt". PT processed with ginger and beef is called "rosy sky-malt". Ferment each product for seven days to become yellowish, and use each product for each disease.
1828 Qing	Shan-lei Zhang (Zhang, 2013)	*Orthodox Interpretation of Materia Medica* (本草正義)	Pungent. No record.	After Northern and Southern dynasties, PT has been processed using ginger or alumen to remove the toxicities of PT. However, by this processing, the drug properties of PT lose, and the author cannot believe these old literatures.
1833 Qing	Shi-tai Yang (Yang, 1958)	*Delving into the Description of Materia Medica* (本草述鉤元)	Pungent and slightly bitter. Warm.	The efficiencies of raw PT are sharp, and those of PTM are soft.<br>Mash PT in the water containing drug adjuvants to make dumplings, wrap them with the leaves of *Broussonetia* sp. or paper, ferment them for seven days, and dry. For several kinds of *phlegm*, use ginger juice. For *wind-phlegmi*, use the decoction of *Gleditschia japonica* hull. For *cold-phlegm* or *moist-phlegm*, use concenrated ginger juice with alumen. For *fire-phlegm* or old *phlegm*, use bamboo sap or *Vitex negundo* var. *cannabifolia* stem juice with small amount of ginger juice. For *phlegm* at skin, use white pepper and bamboo sap. For epilepsia, use cow's bile with concentrated honey. For epilepsia of children, add *Arisaema heterophyllum* rhizome and licorice root. For *phlegm* with *spleen* deficiency, use *Cyperus rotundus* rhizome, *Atractylodes lancea* rhizome, and *Ligusticum sinense* rhizome. For stroke and other cold damages, use mirabilite and white lead frost or use rhubarb. For other severe and lingering illness, use ginger and beef.

1862 Qing	Huan Ling (Zhou, 2012)	*Harm and Benefit in Materia Medica* (本草害利)	Pungent. Warm.	Prepared PT (PPT): Prepare PT by Song dynasty's methods. The drug property is weak. PTM: Roast PT with ginger juice, refine using beef juice, make dumplings, and ferment them. This can treat several kinds of *phlegm*.

References:

Chen, J.M. (2013). Rpt. *Enlightneing Primer of Materia Medica* (本草蒙筌), Beijing: China Traditional Chinese Medicine Publishing House. p. 80–81.

Chen, R.S., and Wu, C.G. (2013). Rpt. *Revised Zhenghe Classified Materia Medica from Historical Classics for Emergency* (重修政和经史证类备用本草), Beijing: China Traditional Chinese Medicine Publishing House. p. 645–648.

Gu, Y.J. (2015). Rpt. *Treasury of Letters on Materia Medica* (本草汇笺), Beijing: China Press of Traditional Chinese Medicine. p. 108–109.

Huang, F.S. (2011). Rpt. *Illumination of Materia Medica* (本草发明), Beijing: Xueyuan Press. p. 76–77.

Huang, G.X. (1979). Rpt: *Seeking Accuracy in Materia Medica* (本草求真), Shanghai: Shanghai Scientific and Technical Publishers. p. 117–118.

Lei, X. (2010). Rpt. *Master Lei's Discourse on Medicinal Processing* (雷公炮制药性解). Beijing: People's Military Medical Press. p. 131–134.

Li, S.Z. (2004). Rpt. *Compendium of Materia Medica* (本草綱目). 2nd. Ed., vol. 2. Beijing: People’s Medicinal Publishing House. p. 979–985.

Li, Z.L. (2007). Rpt. *Origins of Magteria Medica* (本草原始), Eds. J.S. Zheng, W.G. Wang & M.X. Yamng. Beijing: People’s Medicinal Publishing House. p. 148–149.

Li, Z.Z. (2015). Rpt. *Penetrating the Mysteries of Materia Medica* (本草通玄), Beijing: China Traditional Chinese Medicine Publishing House. p. 43–44.

Su, J. (2013). Rpt. *Newly Revised Materia Medica* (新修本草), Taiyuan: Shaanxi People’s Publishing House. p. 16, 232–233.

Tao, H.J. (1997). Rpt. *Collective Commentaries on Classics of Materia Medica* (本草经集注), in: Preface of *Collective Commentaries on Classics of Materia Medica – Dunhuang Manuscript*, Ed. Institute for Buddhist Culture Ryukoku University. Kyoto: Hozokan. p. 246, 264.

Tao, H.J. (2013). Rpt. *Miscellaneous Records of Famous Physicians* (名医别录), Ed. Z.J. Shang. Beijing: China Traditional Chinese Medicine Publishing House. p. 51, 128, 172, 182, 198.

Wang, H.G. (2008). Rpt: *Materia Medica for Decoctions* (汤液本草), Beijing: China Traditional Chinese Medicine Publishing House. p. 73–74.

Xue, J. (2015). *Rpt. Bencaoyueyan* (本草约言). Beijing: China Traditional Chinese Medicine Publishing House. p. 17–18.

Yang, S.T. (1958). Rpt: *Delving into the Description of Materia Medica* (本草述钩元), Shanghai: Science and Technology Health Publishing House. p. 298–301.

Zhang, J.B. (1624). *Orthodox Materia Medica* (本草正). Guanzhou: Chinese Think Tank p. 31–32. https://www.zk120.com/ji/book/1026

Zhang, L. (2011). *Rpt. Encountering with Origin of Herbal Classic* (本草逢原). Beijing: China Medical Science Press. p. 60–61.

Zhang, S.L. (2013). Rpt. *Orthodox Interpretation of Materia Medica* (本草正义), Taiyuan: Shaanxi People's Publishing House. p. 300–302.

Zhao, X.M. (1983). Rpt: *Supplement to Compendium of Materia Medica* (本草纲目拾遗), Beijing: China Traditional Chinese Medicine Publishing House. p. 160–161.

Zhen, Q. (1983). *Treatise on Medicinal Properties* (药性論). Wannan: Wannan Medical College Facultyu of Science Research. p. 33.

Zhou, D.S. (2012). *Rpt. Harm and Benefit in Materia Medica* (本草害利). Taiyuan: Shaanxi People's Publishing House. p. 60–61.
