# Supplementary material for: Historical Study for the Differences of Processing of Pinellia ternata Tuber Between China and Japan
Source: Front Pharmacol. 2022 Jun 20;13:892732. doi: 10.3389/fphar.2022.892732 (PMC9251410; doi:10.3389/fphar.2022.892732)
Supplement: Supplementary file 3 [file Table3.pdf]

Supplementary Table 3. The descriptions about processing of Pinellia Tuber (PT) in medical and medicinal literatures published in Japan.

Year	Author	Literature title	Taste and Property	Descriptions about processing methods
1363	Zennist Yurin (Yurin, 1363)	*Yurin-fukuden-ho* (有林福田方)	No records.	Wash PT in boiling water to remove sliminess, and soak them in hot water seven times. Mix PT with equal amount of ginger, mash them to make dumplings. When it is used, roast and dry them.
1567	Kisan Sugie (Sugie, 1989)	*Yakushu-iroha-sho* (薬種いろは抄)	No records.	Put PT in a bowl and wash them by hand well in water to remove their sliminess. Boil PT in water for half an hour, add ginger in this soup, mash the contents, and then filter them with cloth. Dry the filtrate under sunlight, and roast them.
1581	Dosan Manase (Manase, 1581)	*Hosha-teiyo* (炮炙撮要)	No records.	Wash PT in hot water six or seven times to remove sliminess, and dry them. Processing using ginger is necessary to prescribe PT in the formula to remove their toxicity.
1623	Dosan Manase (Manase, D., 1623a)	*Wamyoshu-narabini-imyo-seizaiki* (和名集並異名製剤記)	No records.	Wash PT in hot water seven times to remove soil and sliminess, and dry them under sunlight. Dress them with ginger and bake it. Alternatively, wash PT seven times, mash them with ginger to make platy round dumplings like rice malt, and ferment for 3 nights. Then, dry them under sunlight to prepare PTM (半夏麹).
1623	Gensaku Manase (Manase, G., 1623b)	*Reiho-yakusho-nodoku* expanded edition (増補霊寳薬性能毒)	Pungent. Neutral. Slightly cold (raw). Warm (Processed).	Wash PT in boiling water, and wash them well seven times to remove soil, sliminess and skin. Grind them in a mortar, add one-tenth weight of ginger, mash and mix them to make platy round dumplings, and keep them one or two days. After becoming yellow, dry them to prepare PTM. When it is used, grind and bake it.
1681	Genri Endo (Endo, 1681)	*Honzo-bengi* (本草弁疑)	No records.	In order to prepare PTM, choose small size unsold PT, soak them in hot water, and mash them to make dumplings. But soaking them, processing with ginger, making PTM can be skipped. Since PT is toxic, soak them in hot water for seven days, mash them with ginger juice which weight is one-fourth of PT to make dumplings, and then ferment them. After yellowish flowers are appeared, they can be used.

1685	Mototomo Shimotsu (Shimotsu, 1685)	*Zukai-honzo* (図解本草)	No records.	Wash PT in hot water to remove skin and grime, and soak them for seven days under daily replacement of hot water to remove sliminess well. Cut and mix with ginger juice, and roast them.<br>PT powder (半夏粉): Grind PT into powder, soak them in hot water containing ginger juice for three days. Remove sliminess in the supernatant, collect the deposits, and dry them.<br>PT cake (半夏餅): Grind PT into powder, mix them with ginger juice to make cake, then dry them under sunlight.<br>PTM: Grind PT into powder, mix them with ginger juice and alumen soup to make dumplings, wrap them with the leaves of *Broussonetia* sp., and ferment them. When yellowish malt flowers are appeared, collect and dry them under sunlight.
1688	Genemon Takenaka (Takenaka, 1688)	*Hiden-yakushoki* (秘伝薬性記)	Pungent. Slightly warm	Mix PT and ginger. Then, the drug property changes into slightly warm.
1696	Unknown (Unknown, 1696)	*Shuchi-sanyo-wage* (修治纂要和解)	Pungent. Neutral.	Soak white and large size PT in boiling water, and wash them well seven times to remove sliminess. Cut and dry them under sunlight. Mix them and ginger juice, then dry and roast them well. Or after the removal of their sliminess, mash them, make dumplings with ginger juice, and ferment them. After being yellowish, dry them under sunlight, then cut and roast them to prepare PTM.
1697	Sekko Masatsugu (Masatsugu, 1697)	*Honzo-wage* (本草和解)	Pungent. Neutral. Slightly cold (raw). Warm (Processed).	Wash PT in boiling water, and wash them well seven times to remove sliminess and skin. Grind them in a mortar, mix them with ginger, mush them to make dumplings, and ferment them. After being yellowish, dry them under moon light to prepare PTM.

1698	Ipposhi Okamoto (Okamoto, 1698)	*Koeki-honzo-taisei* (広益本草大成)	Pungent and bitter. Warm.	Soak PT in hot water, and wash them to remove sliminess and skin. This step is repeated seven times with daily replacement of water. Then, dry them under sunlight, and mix them with ginger juice, dry them again, and roast them. Alternatively, boil PT with ginger juice, alumen soup, or the hull of *Gleditschia japonica*, and dry them.<br>PT powder: Grind PT into powder, soak them in hot water containing ginger juice for three days. Remove sliminess in the supernatant, collect the deposit, and dry them.<br>PT cake: Grind PT into powder, mix it with ginger juice to make cake, then dry under sunlight.<br>PTM: Wash PT, remove sliminess and skin, mash them in a mortar, mix it ginger juice to make flat ball dumplings, wrap them with the leaves of *Broussonetia* sp., and ferment them. When yellowish malt flowers are appeared, collect and dry them under sunlight, then cut and roast them.<br>In order to treat *phlegm*-damps, mix PT with ginger juice and alumen soup. To treat *wind phlegm*, mix them with *Gleditschia japonica* hull soup and ginger juice. To treat *fire phlegm*, mix them with with ginger juice, bamboo juice, or *Vitex negundo* var. *cannabifolia* stem sap. To treat cold *phlegm*, use ginger juice, alumen soup and white mustard powder to make malt.
1702	Jakusui Ino (Ino, 1702)	*Hosha-zensho* (炮炙全書)	Pungent. Neutral.	Wash PT to remove skin. Mix them with reed sap and alumen soup to make dumplings. Wrap them with the leaves of *Broussonetia* sp., and ferment them. When yellowish coats are appeared, collect and dry them. In order *phlegm*-damps, mix PT with ginger juice and alumen soup. To treat *wind phlegm*, mix them with ginger juice *and Gleditschia japonica* hull soup. To treat *fire phlegm*, mix them with with ginger juice, bamboo sap, or *Vitex negundo* var. *cannabifolia* stem sap. To treat cold *phlegm*, mix them with ginger juice, alumen soup and white mustard powder.
1709	Ekken Kaibara (Kaibara, 1709)	*Yamato-honzo* (大和本草)	No records.	Soak PT in hot water seven times. To prepare PTM, roast them excessively. Mix PT with ginger juice to prepare the drugs.

1710	Takatomo Okunishi (Okunishi, 1710)	*Yakushu-shin-seizaiki* (薬種新製剤記)	No records.	Soak PT in hot water, and wash them well seven times to remove sliminess, black things, and sands. Cut and dry them, then mix them with ginger juice, dry them, and roast them well. Alternatively, after washing PT, mash them in a mortar, and make dumplings with ginger juice to ferment them. After being yellowish, dry them under sunlight, then cut and roast them to prepare PTM. Alternatively, after washing PT well, boil them in ginger juice for 3 hours, wash them again, cut and roast them to become black. Since PT is toxic, these processing procedures can reduce their toxicities.
1734	Gyuzan Katsuki (Katsuki, 1734)	*Yakuro-honzo* (薬籠本草)	Pungent. Neutral.	Shi-zhen Li (Li, 2004) said the following. Soak PT in hot water with daily replacement of water for seven days. Dry and cut them, and roast them with ginger juice to prepare the drug. Or grind PT, and soak them in hot water with ginger juice for three days. After removal of the sliminess in the supernatant, collect the deposit, and dry it to prepare "PT powder". Alternatively, grind PT into powder, and mix it with ginger juice to make the cake. Then, dry under sunlight to make "PT cake". Alternatively, grind PT into powder, mix it with ginger juice and alumen soup to make dumplings, wrap them with the leaves of *Broussonetia* sp., and ferment them. When yellowish coats are appeared, collect and dry them under sunlight to prepare PTM.<br>In order to treat *phlegm*-damps, mix PT with ginger juice and alumen soup. To treat *wind phlegm*, mix them with *Gleditschia japonica* hull soup and ginger juice. To treat *fire phlegm*, mix them with with ginger juice, bamboo sap, or *Vitex negundo* var. *cannabifolia* stem sap. To treat cold *phlegm*, use ginger juice, alumen soup and white mustard powder to make malt.
1738	Shuan Kagawa (Kagawa, 1738)	*Ippondo-yakusen* (一本堂薬選)	No records.	Processing methods for PT has been different among literatures in Middle Ages, and in order to reduce its toxicity, prepare its malt, cake, powder using ginger juice, alumen, white pepper, strong vinegar by mushing, roasting, or boiling in ginger juice. When trying to drink the decoction of single PT, some one will find a little different taste from that of simple hot water, but other ones find a little spicy and astringent. This is the result obtained from several trials

				by my apprentices, not by myself alone. Physicians, whether being ancient or modern, have been afraid of the acrid pain at throat based on the experiences of taking a tiny piece of dried PT, as small as a sesame seed, and have been processing it conventionally to decrease its toxicity, in fact with its efficacies altogether. How unwise! They do not know that when we heat PT to prepare the decoction, throat irritation disappears. It is similar that taking raw taro causes throat irritation, but well-cooked taro did not and tasty. Therefore, PT should not be processed.
1771	Todo Yoshimasu (Yoshimasu, 1771)	*Yakucho* (薬徵)	No records.	General physicians process PT using ginger juice, because they fear its toxicities. However, this processing kills the efficiencies of PT, and should not be followed.
1772	Sadaaki Hayashi (Hayashi, 1772)	*Honzo-benmei* (本草弁明)	Pungent. Warm.	Soak PT in water with daily replacement of water for seven days to remove sliminess. Cut and dry them, then mix them with ginger juice and roast them.
1780	Kensai Kato (Kato, 1780)	*Hengyoku-rokuhachi-honzo* (片玉六八本草)	Pungent and bitter. Warm.	Soak PT in hot water for seven days. Grind them into powder, and mix them with ginger juice to make dumplings. Then, dry under sunlight to prepare "PT cake". Alternatively, grind PT into powder, and then soak them in hot water with ginger juice for three days. After the removal of the sliminess in the supernatant, collect the deposit, and dry them to prepare "PT powder". Alternatively, grind PT into powder, mix it with ginger juice and alumen soup to make the cakes, wrap them with the leaves of *Broussonetia* sp., and ferment them. When yellowish coats are appeared, collect and dry them under sunlight to prepare PTM. To strengthen *spleen qi*, use PTM. "Boiled PT (煮半夏)" is prepared by boiling PT with alumen soup, but its effects to relieve *phlegm* is weak. "PT prepared with four kinds of processing (四製半夏)" is prepared using alumen, *Gleditschia japonica* hull, bamboo sap, and ginger juice, and its effectiveness is good.
ca. 1790	Ranzan Ono (Ono, ca. 1790)	*Honzo-kibun* (本草記聞)	No records.	Small size PT that cannot be sold in drug stores, they grind small one to make PTM.

1811	Seikan Shibata<br>(Shibata, 1811)	*Nichiyo-yakuhin-ko*<br>(日用薬品考)	No records.	PTM. There are no imported ones. The products in drug stores are produced by soaking low-quality PT in hot water, grinding them, and making dumplings. They are very rough and have toxicity. Use PTM made by yourself. Processing methods were described in the Compendium of Materia Medica (Li, 2004) in detail.
1824	Ryoan Terashima<br>(Terashima, 1824)	*Wakan-sansai-zue*<br>(和漢三才図会)	Pungent.<br>Slightly cold (raw).<br>Warm (Processed).	To reduce the toxicities of PT, processing using ginger is necessary. Soak PT in hot water with daily replacement of water for seven days to remove remove sliminess, then dry, cut, and roast them. Grind them into powder, and mix it with ginger juice to make dumplings. Then, dry under sunlight to prepare "PT cake". Grind them into powder, and then soak them in alumen soup with ginger juice to make dumplings. Wrap them with the leaves of *Broussonetia* sp., and ferment them. When yellowish coats are appeared, collect and dry them under sunlight to prepare PTM.
1840	Hisakata Naito<br>(Naito, 1840)	*Koho-yakuhin-ko*<br>(古方薬品考)	Pungent.<br>Neutral.	Past people, the appearance of the effectiveness was faster in using PT with fresh or dried ginger. However, do not kill the toxicity of PT using ginger. Since raw dried PT causes irritation in throat and tong of patients, roast them well when patients take the powdered prescriptions. Only unprocessed raw PT has their effectiveness to rescue the dying patients described in *Handbook of Prescriptions for Emergency* (Ge, 2016).
1847	Ranzan Ono<br>(Ono, 1847)	*Jutei-honzo-komoku-keimo*<br>(重訂本草綱目啓蒙)	No records.	Mash small size PT in hot water. Make the dumplings to prepare PTM. Alternatively, mix PT with ginger juice and alumen soup, the ferment them. When yellowish coats have appeared, it is called as PTM.
1850	Tadahiro Kitamura<br>(Kitamura, 1850)	*Shokan-yakugi*<br>(傷寒薬議)	Pungent.<br>Neutral.	Wash PT in hot water for ten times. To reduce the toxicities of PT, processing using ginger is necessary.
1929	Naotaro Isshiki<br>(Isshiki, 1929)	*Wakanyaku-no-ryohi-kanbetsuho-oyobi-choseiho*<br>(和漢薬の良否鑑別法及調製方)	No records.	Soak raw PT in hot water six or seven times to remove sliminess. Then put them in a mortar, add one tenth of the weight of raw ginger, and mash them. After making flat ball dumplings, ferment them for one or two days. Then, dry them under sunlight to make PTM.

1939	Keisetsu Otsuka (Otsuka, 1939)	Toxicity of PT	No records.	Shi-zhen Li said that PT had toxicities (Li, 2004), however, I don't think this means that it is not poison. I think that it makes no sense to get rid of the toxicities of PT. The Korean physician I have known said that PT has two effects, to stop and to cause vomiting. Therefore, only the effect of causing vomiting must be removed. Thus, it is said that PT should be soaked in the water after washing rice overnight. It is said that by the toxicities of PT, it should not be used for babies. However, I do not believe these traditions.
1980	Tsuneo Namba (Namba, 1979)	Colored illustrations of wakan-yaku (the crude drugs in Japan, China and the neighbouring countries) (原色和漢薬図鑑)	Pungent. Warm.	PT should be used to treat *cold phlegm* or *wind phlegm*. In order to treat dried *phlegm* or *hot phlegm*, PT processed using baboo sap, *Scutellaria baicalensis* root, or ginger juice.

References:

Endo, G. (1681). *Honzo Bengi* (本草弁疑). Tokyo: National Diet Library Digital Collections. p. 24–25. https://dl.ndl.go.jp/info:ndljp/pid/2607109

Hayashi, S. (1772) *Honzo Benmei* (本草弁明), Tokyo: National Diet Library Digital Collections. p. 23–25. https://dl.ndl.go.jp/info:ndljp/pid/2536779

Ino, J. (1702). *Hosha Zensho* (炮炙全書). Tokyo: National Diet Library Digital Collections. p. 40. https://dl.ndl.go.jp/info:ndljp/pid/2605870

Isshiki, N. (1929). *Wakanyaku no Ryohi Kanbetsuho Oyobi Choseiho* (和漢薬の良否鑑別法及調製方). Tokyo: Kyorin Publishing. p. 40–41. https://dl.ndl.go.jp/info:ndljp/pid/931606

Kagawa, S. (1738). *Ippondo Yakusen* (一本堂薬選). Tokyo: National Diet Library Digital Collections. p. 32–33. https://dl.ndl.go.jp/info:ndljp/pid/2606390

Kaibara, E. (1709). *Yamato Honzo* (大和本草). Tokyo: National Diet Library Digital Collections. p. 22. https://dl.ndl.go.jp/info:ndljp/pid/2605899

Kato, K. (1780). *Hengyoku Rokuhachi Honzo* (片玉六八本草). Tokyo: National Diet Library Digital Collections. p. 23–25. https://dl.ndl.go.jp/info:ndljp/pid/2610496

Katsuki, G. (1734). *Yakuro Honzo* (薬籠本草). Tokyo: National Diet Library Digital Collections,. p. 30–35. https://dl.ndl.go.jp/info:ndljp/pid/2605195

Kitamura, T. (1850). *Shokan Yakugi* (傷寒薬議). Tokyo: National Diet Library Digital Collections. p. 35–37. https://dl.ndl.go.jp/info:ndljp/pid/2536449

Manase, D. (1581). *Hosha Teiyo* (炮炙撮要). Tokyo: National Diet Library Digital Collections. p. 21. https://dl.ndl.go.jp/info:ndljp/pid/2535841

Manase, D. (1623a). *Wamyoshu narabini Imyo Seizaiki* (和名集並異名製剤記). Tokyo: National Diet Library Digital Collections. p. 7–8. https://dl.ndl.go.jp/info:ndljp/pid/2575934

Manase, D. (1623b). *Reiho Yakusho Nodoku Expanded Edition* (増補霊寳薬性能毒). Tokyo: Waseda University Library. p. 20–22. https://www.wul.waseda.ac.jp/kotenseki/html/bunko31/bunko31 e1590/index.html

Masatsugu, S., 1697. Honzo Wage (本草和解), Tokyo: National Diet Library Digital Collections. p. 26–29. https://dl.ndl.go.jp/info:ndljp/pid/2607215

Naito, H. (1840). *Koho Yakuhin Ko* (古方薬品考). Tokyo: National Diet Library Digital Collections. p. 137–138. https://dl.ndl.go.jp/info:ndljp/pid/2536679

Namba T, (1979) Coloured illustrations of wakan-yaku (原色和漢薬図鑑), Hokuryukan, Tokyo, p. 46–47.

Okamoto, I. (1698). *Koeki Honzo Taisei* (広益本草大成). Tokyo: National Diet Library Digital Collections. p. 23–27. https://dl.ndl.go.jp/info:ndljp/pid/2606062

Okunishi, T. (1710). *Yakushu Shin Seizaiki* (薬種新製剤記). Tokyo: National Diet Library Digital Collections. p. 10. https://dl.ndl.go.jp/info:ndljp/pid/2606704

Ono, R. (ca. 1790). *Honzo Kibun* (本草記聞). Tokyo: National Diet Library Digital Collections. p. 20–21. https://dl.ndl.go.jp/info:ndljp/pid/2605532

Ono, R (1847) *Jutei Honzo Komoku Keimo* (重訂本草綱目啓蒙). Tokyo: National Diet Library Digital Collections.vol. 6, p. 24–25. https://dl.ndl.go.jp/info:ndljp/pid/2606063

Otsuka, K. (1939). Toxicity of Pinellia Tuber. *Kampo and Kanyaku* 1**,** 806.

Shibata, S. (1811). *Nichiyo Yakuhin Ko* (日用薬品考), Tokyo: National Diet Library Digital Collections. p. 18. https://dl.ndl.go.jp/info:ndljp/pid/2536868

Shimotsu, M. (1685). *Zukai Honzo* (図解本草), Tokyo: National Diet Library Digital Collections. p. 27–28. https://dl.ndl.go.jp/info:ndljp/pid/2606815

Sugie, K., 1989. Rpt. Yakushu Iroha Sho (薬種いろは抄) in: Setsuyo-shu, Kokujin, Yakushu Iroha Sho. Ed. Kobayashi, K. Osaka: Seibundo. p. 153.

Takenaka G, (1688) Hiden Yakushoki (秘伝薬性記), Tokyo: National Diet Library Digital Collections. p. 54–55. https://dl.ndl.go.jp/info:ndljp/pid/2536944

Terashima, R. (1824). *Wakan Sansai Zue* (和漢三才図会). Tokyo: National Diet Library Digital Collections. p. 20–21. https://dl.ndl.go.jp/info:ndljp/pid/2609003

Unknown, 1696. Shuchi Sanyo Wage (修治纂要和解). Tokyo: Public Interest Incorporated Foundation KENIKAI. p. 18–19.

https://kotenseki.nijl.ac.jp/biblio/100248429/viewer/1

Yoshimasu, T. (1771). *Yakucho* (薬徴). Tokyo: National Diet Library Digital Collections. p. 41. https://dl.ndl.go.jp/info:ndljp/pid/2606247

Yurin, Z. (1363). *Yurin Fukuden Ho* (有林福田方). Kyoto: Kyoto University Rare Materials Digital Archive. p. 20. https://rmda.kulib.kyoto-u.ac.jp/item/rb00005522
